# Changes of plasma fibronectin and fibronectin-fibrin complexes in dams of stillborn dairy calves

**DOI:** 10.1186/s13620-020-00171-1

**Published:** 2020-08-09

**Authors:** Paulina Jawor, Dorota Krzyżanowska-Gołąb, Joanna Bajzert, Tadeusz Stefaniak, Iwona Kątnik-Prastowska

**Affiliations:** 1 Department of Immunology, Pathophysiology and Veterinary Preventive Medicine, Wroclaw University of Environmental and Life Sciences, 50-375 Wrocław, Poland; 2 Department of Chemistry and Immunochemistry, Wroclaw Medical University, 50-369 Wrocław, Poland

**Keywords:** Fibronectin, Fibrinogen, Fibronectin-fibrin complexes, Calf perinatal mortality, Dairy cow

## Abstract

**Background:**

Fibronectin (FN) is a large (450–500 kDa), multidomain and multifunctional glycoprotein existing in mammalian tissues. Some fibronectin (FN) molecular forms might be involved in biological processes occurring within the perinatal period, such as tissue remodeling, coagulation, and repair.

**Results:**

In this study fibronectin (FN) and fibrinogen (Fb) concentrations and FN-fibrin complexes occurrence and its relative amounts with increasing high molecular masses were respectively determined by ELISA, heat precipitation, and SDS-agarose-immunoblotting methods. Plasma samples from three groups of dams with: 1) singleton stillborn calf without or with negligible autolytic changes in internal organs (DSBn), 2) singleton stillborn calf with advanced autolytic changes in internal organs (DSBa), 3) singleton live-born control calf (DC), and 4) a group of cows during mid to late lactation (LC) were analyzed. Maternal plasma FN concentration in the DSBn and DSBa groups was significantly lower than in the LC group. The plasma samples of DSBa showed a significantly lower FN concentration than in the DC group. Plasma Fb concentration was significantly higher in the DSBa and DSBn, than in the LC group. FN immunoblotting of the cow plasma samples revealed, besides an FN-dimer band, the presence of supramolecular FN-fibrin bands corresponding to FN-fibrin complexes with increasing molecular masses: up to 5 bands from 750 kDa to 1900 kDa in the DSBn and DSBa plasma samples, two bands of 750 and 1000 kDa in the DC group, and only the smallest one of 750 kDa in the LC group.

**Conclusions:**

The observed low FN concentration and occurrence of supramolecular FN-fibrin complexes (1000 kDa and more) in the maternal plasma comparing to cows in lactation might have been associated with periparturient changes in tissues. The presence in maternal plasma of high-molecular FN-fibrin complexes (1300–1900 kDa) arouse the question if this is the consequence of calf perinatal mortality.

## Background

The perinatal period is the most hazardous time in the life of cattle. Bovine perinatal mortality (PM) may be defined as calf death at full-term pregnancy (≥260 days), before, during, or within 48 h of calving [1]. Incidence of perinatal mortality in the majority of countries vary between 4 and 7% [2]. Apart from calf loss, cows that give birth to a stillborn calf have a significantly increased risk of culling/death throughout the lactation period and exhibit an increase of 88 days in median days open compared with cows that had live calves [3]. Cows also experience different degree of systemic periparturient inflammation [4].

Fibronectin (FN) is a large (450–500 kDa), multidomain and multifunctional glycoprotein existing in mammalian tissues in many molecular iso- and glyco-forms which are present in blood plasma (pFN) in a soluble form, and in the extracellular matrix (ECM) and on the surface of cells as an insoluble cellular molecule [5–7]. Plasma FN originating from synthesis in the liver can act as a non-specific opsonin which facilitates the removal of tissue debris (including collagen and fibrin) by macrophages and neutrophils [8]. The basic form of fibronectins is a dimer, made of two identical or nearly identical disulphide-bonded polypeptide chains (220–250 kDa) composed of a series of short modules linked and organized into several structurally and functionally independent domains which bind specifically natural FN ligands. FN has a great ability to form complexes with other molecules that are engaged in processes associated with cell–matrix interactions [6, 7, 9, 10]. Inflammation-associated tissue condition, local transient hypoxia, and pH changes stimulate leakage of plasma FN to tissue, where it changes the conformation from globular to fibrillar. Tissue damage and an enhanced thrombotic condition activates the coagulation cascade in which fibrinogen (Fb) molecules are converted to fibrin and into its circulating polymers with an increasing number of fibrin subunits. Transglutaminase XIIIa catalyzes covalent cross-linking of FN to the carboxyl αC-domain of fibrin [11]. The crosslinked FN-fibrin polymers form a lattice of provisional matrix in damaged tissue [6, 10, 12–14], and moreover they may occur in biological fluids [15–18]. The circulating soluble FN-fibrin complexes with molecular masses from around 750 kDa to 2100 kDa were revealed by Kątnik-Prastowska’s group in puerperal plasma samples in human patients with some diseases, especially when inflammation was present, but rarely in healthy individuals [15–18].

Assuming that some molecular FN forms can be directly or indirectly engaged in the inflammatory response during periparturient and repair process during postparturient period, we hypothesized that the changes in FN concentration and occurrence of FN-fibrin complexes in maternal plasma can be associated with perinatal mortality of calves. In this study the FN and Fb concentrations, presence of FN-fibrin complexes and their relative amounts were determined in the maternal plasma samples with singleton stillborn calves confirming the absence or presence of significant autolytic changes by necropsy in comparison to dams giving birth to live calves.

## Results

### FN and Fb concentrations

As shown in Table 1, the FN concentration in the DSBa group was significantly lower than in the DC group. No difference in FN concentration was observed between the maternal plasma samples of DSBa and DSBn groups. However, the FN concentration in DSBa and DSBn groups was significantly lower than that in the LC group.

**Table 1:** Fibronectin and fibrinogen concentrations in maternal plasma in relation to perinatal mortality of calves

Parameter	Mean ± SD median (25th–75th percentile) values of concentration in the plasma groups:			
	DSBa *n* = 47	DSBn *n* = 45	DC *n* = 21	LC *n* = 14
FN mg/L	40.0 ± 17.5	47.5 ± 20.5	57.5 ± 13.1	71.7 ± 15.2
	34.7 (23.9–55.4)^A^	45.2 (29.4–63.5)^AC^	59.2 (51.7–64.2)^BC^	74.1 (62.3–83.0)^B^
Fb g/L	4.9 ± 1.7	4.9 ± 1.5	4.9 ± 1.5	3.6 ± 1.1
	5.1 (3.7–5.9)^a^	4.9 (3.7–5.9)^a^	4.3 (3.7–6.2)	3.4 (2.9–3.9)^b^

A, B, C, D – values in rows with different letters differ significantly *P* < 0.01.

a, b, c, d– as above for *P* < 0.05.

DSBa – dams of singleton stillborn calves which internal organs showed significant autolytic changes, DSBn – dams of singleton stillborn calves without or with negligible autolytic changes in internal organs, DC – dams of live-born calves, LC – cows during mid to late lactation.

Fb concentration (Table 1) in the DSBa and DSBn groups was significantly higher than in the LC group.

### FN degradation products

The electrophoresis of plasma samples in SDS-polyacrylamide gel under reducing conditions and subsequent immunoblotting with anti-FN monoclonal antibody did not show the presence of FN fragments.

Occurrence and relative amount of plasma FN-fibrin complexes.

**Fig. 1:**
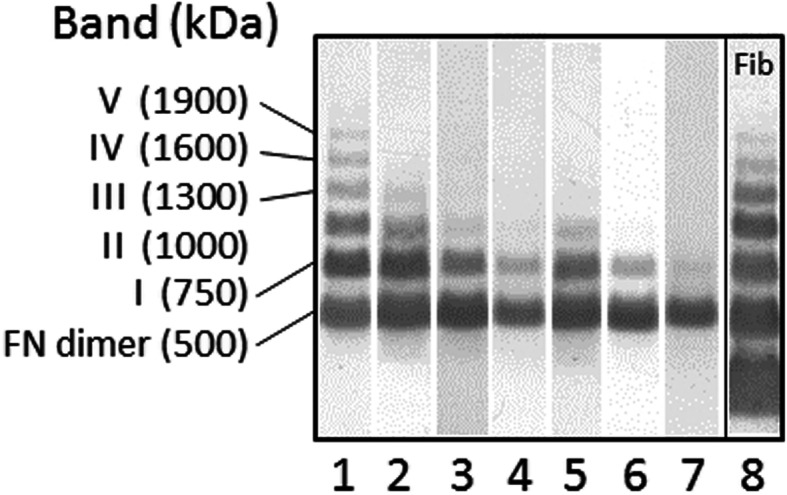
Immunoblotting patterns of FN-fibrin complexes in plasma samples of dams and cows in mid to late lactation. Representative pattern agarose-electrophoresis separated plasma FN of dams of stillborn calves (lanes 1–4), dams of live-born calves (lanes 5–6), and cows in mid to late lactation (lane 7). Lane 8 – the plasma sample used in lane no. 1 developed with rabbit antiserum against human fibrinogen

FN-SDS-agarose immunoblotting of bovine plasma revealed the presence of up to six bands with decreasing electrophoretic mobilities (Fig. 1). The band of ~ 500 kDa corresponded to a dimer of FN, whereas the bands of ~ 750 kDa, ~ 1000 kDa, ~ 1300 kDa, ~ 1600 kDa, and ~ 1900 kDa recognized by anti-FN monoclonal antibody and anti-fibrinogen polyclonal antibodies corresponded to the supramolecular FN-fibrin complexes numbered I–V, respectively.

In the maternal plasma samples of DSBa and DSBn groups up to 5 bands of the FN-fibrin complexes with increasing molecular masses of 750–1900 kDa were detected (Fig. 1, Table 2). In contrast, up to two bands of the FN-fibrin complexes with the lowest molecular masses of 750 kDa and 1000 kDa and exclusively the band of 750 kDa were observed in the DC and LC groups, respectively (Fig. 1, Table 2).

**Table 2:** Occurrence and relative amounts of FN-fibrin complexes in plasma of dams and cows during lactation

Group	Frequency of occurrence relative amount (%) median quartiles (25th and 75th percentiles) of FN-fibrin complexes				
	I (~ 750 kDa)	II (~ 1000 kDa)	III (~ 1300 kDa)	IV (~ 1600 kDa)	V (~ 1900 kDa)
DSBa (*n* = 39)	100 29.6 ± 5.1^A^ 30.8 (26.2–33.1)	89.7 7.9 ± 5.2^A^ 7.4 (4.2–13.1)	46.1 1.4 ± 2.6 0.0 (0.0–1.4)	12.8 0.6 ± 2.0 0.0 (0.0–0.0)	7.7 0.2 ± 0.9 0.0 (0.0–0.0)
DSBn (*n* = 37)	100 28.3 ± 5.5^A^ 29.2 (26.4–32.1)	91.9 7.8 ± 6.3^A^ 5.9 (3.6–12.0)	27.0 1.3 ± 3.0 0.0 (0.0–0.8)	13.5 0.4 ± 1.3 0.0 (0.0–0.0)	5.4 0.1 ± 0.8 0.0 (0.0–0.0)
DC (*n* = 15)	100 27.6 ± 3.8^A^ 28.9 (23.7–31.4)	86.7 4.2 ± 2.4^a^ 4.2 (3.0–5.2)	0	0	0
LC (n = 14)	100 10.9 ± 1.8^B^ 11.1 (9.5–12.6)	0^Bb^	0	0	0

A, B, C, D – values in columns with different letters differ significantly *P* < 0.01.

a, b, c, d– as above for *P* < 0.05.

As shown in Table 2, the 750-kDa FN-fibrin complex I was present in all maternal plasma samples and its relative amount was similar in the DSBa (29.6 ± 5.1%), DSBn (28.3 ± 5.5%), and DC (27.6 ± 3.8%) groups, and the values were significantly higher than in the LC group (10.9 ± 1.8%). The 1000-kDa band of FN-fibrin complex II was present in the majority of samples (> 86%) and the relative amounts in DSBa (7.9 ± 5.2%), DSBn (7.8 ± 6.3%), and DC (4.2 ± 2.4%) groups were significantly higher than in the LC group. The 1300-, 1600- and 1900-kDa bands of FN-fibrin complexes III-V were absent in the DC and LC groups, but were detected in 46.1, 12.8, and 7.7% of samples of DSBa and 27.0, 13.5, and 5.4% of samples of DSBn groups respectively. Their relative amounts were similar and low (< 1.5%) in DSBa and DSBn groups and amounts were decreasing with the increasing molecular masses of the bands.

The bands of plasma FN forms (see Fig. 1) were revealed by SDS-agarose electrophoresis followed by immunoblotting (see methods). The bands were scanned and analyzed by densitometry using GelScan V6.0 (BioSciTec GmbH, Frankfurt/Main, Germany). Data are presented as the frequency of occurrence (percentage of samples containing the band; first line). The relative amount (%) of the FN band is the percentage of the total number of pixels found in the analyzed band in electrophoresis path of individual sample in relation to the total number of pixels found for all bands of the sample path (second line). Data are expressed as mean value ± SD (second line), median and quartiles (25th and 75th percentiles) (third line).

DSBn – dams of perinatal mortality calves without or with negligible autolytic changes in internal organs, DSBa – dams of perinatal mortality calves with significant autolytic changes in internal organs, DC – dams of alive calves, LC – cows during mid to late lactation. For details see Materials and methods.

Correlation between the occurrence of FN-fibrin complexes and plasma fibrinogen levels.

The concentration of Fb in the LC group and the relative amount of 750-kDa FN-fibrin complex I were significantly correlated (0.8, *P* ≤ 0.001). In contrast, there was no significant correlation between Fb concentration and the relative amount of FN-fibrin complexes in the DSBa, DSBn and DC groups.

## Discussion

Our results show for the first time that the low plasma FN concentration and presence of two and up to five bands of the high molecular FN-fibrin complexes, with increasing molecular masses were associated with calving of live- and stillborn calves, respectively. In contrast, the occurrence of single FN-fibrin complex in the plasma with the smallest molecular mass of 750 kDa was found in plasma samples from cows at mid to late lactation.

In cattle during the perinatal period depression of dry matter intake during 2 to 3 weeks is well known [19]. Moreover, before parturition nutrient requirements for cows are increasing, causing the inability to cover nutritional requirements, which may lead to a negative energy balance disturbing metabolism of proteins [19, 20]. We found a significantly lower concentration of FN in maternal plasma samples of calves with significant autolytic changes compared with those of live-born calves and cows during lactation. This fact may be related to more intensive pFN leakage from blood to inflamed and deteriorated tissue [21]. In tissue, FN might be used for several cellular and molecular actions, leading to maintaining the balance disturbed by the parturition process and/or pathological processes caused by remaining dead fetuses in the uterus before delivery. Plasma-derived FN can be used to opsonize and remove cellular debris and other insoluble molecules through phagocytosis [6, 8]. Moreover, plasma FN molecules are known to have the ability to prevent platelet aggregation and thrombus formation [22, 23], reduce the area of inflammation, accelerate healing, and are essential in fibrillogenesis taking part in ECM remodeling: FN is incorporated into the fibrillar network of provisional ECM, playing a role in an early phase of controlled repair of wounds [6, 24–26]. On the other hand, the parturition process provokes enhanced synthesis of inflammatory cytokines in cattle [27] and humans [28], which initiate physiological delivery-related inflammatory reactions which in turn stimulate synthesis of some acute-phase proteins, including FN and Fb [29–31]. A high fibrinogen concentration is also reported at the time of delivery and placental expulsion [31]. Activation of the coagulation cascade by an inflammatory agent(s) leads to formation of fibrin from fibrinogen, which easily forms with pFN macromolecular complexes, which has been reported to occur in plasma of human patients who suffer from some inflammatory diseases [16–18, 32] and in cynomolgus monkey plasma after treatment with toxic high doses of recombinant clotting factor XIII [33].

In women after delivery of a healthy newborn at term the plasma FN concentration increases and in most samples three bands of supramolecular plasma FN-fibrin (750, 1000 and 1300 kDa) were revealed in maternal plasma. Such high molecular complexes were not observed in plasma of non-pregnant women [30].

The results from the present study show that in cows which delivered alive calves (DC group) up to two (750-kDa and 1000-kDa) bands of the FN-fibrin complexes were present in plasma. In cows during mid to late lactation only 750-kDa bands were present. On the other hand, in the maternal plasma (DSBa, and DSBn groups) the presence of up to five supramolecular plasma FN-fibrin bands from 750 kDa to 1900 kDa was confirmed. It may be concluded, that the presence of multiple, high molecular FN-fibrin complexes is maternal plasma is associated with cases of calves perinatal mortality. The differences in above described FN-fibrin pattern, lack of correlation between Fb concentration and the relative amount of FN-fibrin complexes in the dams and in contrary a high correlation between Fb concentration and the relative amount of FN-fibrin complex I in the LC group suggest different roles of FN and FN-fibrin complexes at calving and during lactation.

The FN-fibrin complexes, as suggested by Wang and Ni [23], have an opposite role to free pFN, i.e., they show prothrombotic activities supporting platelet aggregation and stabilizing thrombus growth. The fibrils of FN-fibrin take part in the repair process: they are incorporated into the fibrillar network of the ECM in the injured tissue, and in consequence the tissue lesion is repaired by the wound healing process [6, 25, 26]. Lis-Kuberka et al. [30] suggested that the occurrence and high relative amount of delivery-associated FN-fibrin complexes in puerperal plasma reflect intensive remodeling and repair processes after delivery, and moreover might be associated with the physiological adaptive mechanisms reducing the risk of hemorrhage.

The present results show that high concentrations of fibronectin and presence of only one FN-fibrin complex with the smallest molecular mass of 750 kDa in a relatively low amount in the plasma of mid to late lactation cows, might be associated with physiological low-grade activation of the coagulation cascade during milk production in mid to late lactation. Contrary, low FN concentration and presence of 750-kDa and 1000-kDa FN-fibrin complexes in maternal plasma may correspond to temporary hemostasis imbalance associated with remodeling and repair processes intensified after parturition, and physiological adaptive mechanisms.

## Conclusions

The observed low FN concentration and occurrence of supramolecular FN-fibrin complexes (1000 kDa and more) in the maternal plasma comparing to cows in lactation might have been associated with periparturient changes in tissues. The presence of high-molecular FN-fibrin complexes (1300–1900 kDa) in maternal plasma arouse the question if this is the consequence of calf perinatal mortality. To date, no observation of FN-fibrin complexes has been carried in the plasma of pregnant women’s. These complexes have been observed in the plasma of women after delivery. Their occurrence is rather interpreted as associated with the physiological adaptive mechanisms reducing the risk of hemorrhage and intensive remodeling and repair processes after delivery [30]. It remains unknown whether in some pathological conditions it may occur even before birth. We can assume that in the case of intrauterine fetal death FN-fibrin complexes may appear in blood plasma, they could then be a marker of intrauterine death. This requires further research conducted on the dams in the end of pregnancy up to calving in order to observe when those complexes are appear in the plasma.

## Material and methods

The experimental design was approved by II Local Ethics Committee in Wroclaw (permission numbers 23/2012, 58/2014, 60/2014).

Animals and sampling procedure.

The study was carried out on 92 dams of singleton stillborn calves and 21 cows that gave birth to 21 alive, healthy calves (DC). Cows gave birth after a gestation length of ≥260 days. The material was collected from November 2013 to June 2015 in 25 Holstein-Friesian herds from South-West Poland (1–1037 cows/herd; median 90 cows/herd) and the mean (SD) herd calving interval and previous lactation milk yield were 419 [32] days and 8879 (1533) kg/cow/305 DIM, respectively. A free-stall housing system was present in 12 herds, a tie-stall housing system was present in 11 herds, and in two herds both systems were used. In 15 herds cows were fed with total mixed ration and in 10 herds feedstuffs were fed separately. Fourteen cows in the mid to late lactation (LC, 120–400 days in milk) originated from 2 herds enrolled in the study. Seventy-nine percent of these cows were pregnant.

The blood was aseptically taken from the coccygeal vein of all recruited cows into tubes with 9 mL of lithium-heparin (Sarstedt, Germany, cat no. 02.1065.001) and in 10 mL serum separator tubes (MEUS Srl, Piove di Sacco, Italy, cat no. 18203). Additionally from 76 dams of singleton stillborn calves, 15 DC and all LC cows, blood was withdrawn into tubes containing 4.3 mL of sodium citrate 3.2% (1:10) (Sarstedt, Germany, cat no. 04.1922.001). The blood from the dams of stillborn calves was taken when the calf was collected. Necropsies of calves were performed 8 ± 3 h after calving. Based on calf tissue necropsy changes, dams of singleton stillborn calves were divided into two subgroups: DSBa – dams of stillborn calves (*n* = 47) whose internal organs exhibited advanced autolysis during necropsy (i.e., marked or generalized hemoglobin-stained imbibition and/or liquefied parenchyma of kidney, spleen, liver, and brain) and DSBn – dams of stillborn calves (*n* = 45) whose internal organs (i.e. kidney, spleen, liver and/or brain) showed no or negligible autolysis during necropsy. The blood from the mothers of live-born calves was collected within 9 h of calving. All blood samples were centrifuged within 4 h (14 min at 1860 g at room temperature (RT). The separated plasma samples were aliquoted and frozen at − 80 °C until analysis. Before analyses the plasma samples were thawed at room temperature for 60 min and centrifuged for 5 min at 8000 g.

### Fibronectin concentration

FN concentration was determined by sandwich-type solid-phase enzyme-linked immunoassay (ELISA) as described previously [34] with slight modification. A specific monoclonal antibody (M002, TaKaRa, Bio Inc., Japan) reacting with the cell-binding domain of human and bovine fibronectin FN 12–8, diluted 1:1000 with Tris-buffered saline, pH 7.5 (TBS), was used as a capture antibody on a microtiter plate (439,454, Maxisorp Nunc-ImmunoPlate, Thermo Scientific, Denmark) in a volume of 100 μl per well, to bind FN from the sample. Coating was provided for 2 h at 36 °C. The plasma samples were diluted with TBS containing 0.05% Tween 20 (TBS-T) 1000- and 2000-fold. Hundred μl/well of plasma samples from lithium-heparin tubes were incubated for 1 h at 36 °C. The amount of FN bound was quantified by rabbit anti-bovine FN polyclonal antibodies (ab23752, Abcam, UK) followed by peroxidase conjugated goat anti-rabbit immunoglobulins (A4914, Sigma-Aldrich, USA). Both antibodies were diluted 2500-fold in TBS-T, applied 100 μl per well and incubated for 1 h at 36 °C. After each step, a plate was washed with 250 μl per well of TBS-T four times for 3 min at room temperature. A colorimetric reaction was developed using 0.05% o-phenylenediamine dihydrochloride/0.006% H_2_O_2_ as the enzyme substrate, 200 μl per well, incubating for 0.5 h in the dark at room temperature. The reaction was stopped by adding 50 μl of 12.5% H_2_SO_4_ per well. Absorption of the product was measured in a Stat Fax 2100 Microplate Reader (Awareness Technology Inc., USA) at 492 nm with 630 nm as a reference filter. A bovine plasma FN preparation (F4759, Sigma-Aldrich, USA) in amounts from 1.56 ng/well to 25.0 ng/well was used as a standard. Intra- and inter-assay coefficients of variation were 8.9 and 14.8%, respectively.

### Western blotting

To the citrate plasma samples dissociation buffer (Laemmli 2x Concentrate Sample Buffer (Bio-Rad #1610737) with 5% 2-mercaptoethanol) was added and samples were heated at 60 °C for 15 min and centrifuged for 10 min at 10000 x g. SDS-PAGE of plasma samples (~ 2 μl plasma per lane) was done in 7% polyacrylamide gel. After separation the proteins were transferred onto nitrocellulose membranes (10,600,002, GE Healthcare Amersham Protran) using a Trans Blot Turbo system (Bio-Rad semi-dry chamber; transfer conditions: 25 V const., 1.5 mA, 65 min). A discontinuous buffer system was used and 1% SDS was added to the transfer buffer (25 mM Tris, 192 mM glycine, 20% methanol; pH = 8.3) on the gel/cathode side. The membrane was blocked using 3% skim milk in TBS buffer (pH = 7.3; blocking conditions: 180 min at 37 °C). The first antibody was a mouse monoclonal antibody to the fibronectin clone FN12–8 (M002, TaKaRa, Bio Inc., Japan) was used. The membrane was incubated with the primary antibody (diluted 1:2000 in TBST buffer) overnight at 4 °C. The second antibody was a goat anti-mouse IgG HRP-conjugate (A-9044 Sigma-Aldrich, USA) was applied (diluted 1:4000 in TBST buffer). The membrane was incubated with the secondary antibody for 90 min at RT. The reaction was developed with 4-chloro-1-naphthol as the substrate. The fibronectin standard (pro-059-b, Prospec, Israel) was used as a positive control (~ 500 ng/lane).

### Separation of fibronectin in SDS-agarose electrophoresis and fibronectin immunoblotting

Supramolecular forms of bovine plasma FN-fibrin complexes were detected by SDS-agarose electrophoresis followed by immunoblotting (FN-SDS-AGE immunoblotting) according to the method described earlier [15] with minor modifications, i.e. using monoclonal anti-bovine and -human FN antibody FN12–8 (M002, TaKaRa Bio Inc., Japan) diluted 1:2000. Only citrate plasma samples were analyzed. Briefly, the samples of 1.5 μl of bovine citrate plasma were subjected to SDS-agarose electrophoresis followed by capillary transfer on PVDF membrane (162–0177, Immun-Blot PVDF, Bio-Rad, USA). Next, after a blocking step with 3% non-fat milk in TBS buffer, the membrane was incubated with primary antibody as given above. After washing (TBS buffer containing 0.05% Tween 20) and incubating with peroxidase-conjugated goat anti-mouse immunoglobulins (A0168, Sigma-Aldrich, USA) diluted 1:5000, the color reaction was done with 3,3′-diaminobenzidine. For immunoblotting to detect the complexes containing fibrin, rabbit antiserum to human fibrinogen (55,124, MP Biomedicals, USA) cross-reacting with bovine fibrinogen (diluted 1:2000) and peroxidase-conjugated goat anti-rabbit IgG (diluted 1:20000) (A4914, Sigma-Aldrich, USA) as a secondary antibody were used. The bands were scanned and analyzed by densitometry using GelScan V6.0 (BioSciTec GmbH, Germany). Molecular masses were estimated on the basis of electrophoretic mobility of von Willebrand factor multimers, as described previously [15].

### Fibrinogen concentration

Fibrinogen (Fb) was analyzed in lithium-heparin plasma using a heat precipitation method, following procedures previously described [35].

### Statistical analysis

Statistical analyses were conducted using STATISTICA 12.5 (StatSoft Inc.,

Tulsa, OK, USA). Data of FN and Fb concentrations and relative amounts of FN-fibrin complexes showed abnormal distribution in the Shapiro-Wilk test. Statistical analysis for Fb, FN concentration and relative amount of FN-fibrin complexes in plasma was performed using the Kruskal-Wallis test, a non-parametric analysis of variance (ANOVA). Data are presented as means ± standard deviations (SD), medians and quartiles (25th and 75th percentiles). Spearman’s correlation coefficient was calculated for fibrinogen concentration and FN-fibrin complexes. *P* values lower than 0.05 were regarded as significant in each statistical test.

## Data Availability

The datasets used and analyzed during the current study are available from the corresponding author on reasonable request.

## Abbreviations

FN: Fibronectin
Fb: Fibrinogen
DSBn: Dams of singleton stillborn calf without or with negligible autolytic changes in internal organs
DSBa: Dams of singleton stillborn calf with advanced autolytic changes in internal organs
DC: Singleton live-born control calf
LC: Cows during mid to late lactation
pFN: Blood plasma fibronectin
ECM: Extracellular matrix
